# Nursing care of a patient with severe pneumonia complicated with multiple disorders under a multidisciplinary team: A long-term case report

**DOI:** 10.1097/MD.0000000000042435

**Published:** 2025-05-09

**Authors:** Zhenzhen Yuan, Yinhao Yang, Qun Liu, Bolun Zhang, Xingxing Wang, Shiqun Huang, Wenli Ma

**Affiliations:** a Department of Critical Care Medicine, Xiangyang No.1 People’s Hospital, Hubei University of Medicine, Xiangyang, Hubei, China.

**Keywords:** critical care nursing, multidisciplinary team, severe pneumonia

## Abstract

**Rationale:**

Severe pneumonia is a critical stage in the course of pneumonia, which is accompanied by respiratory failure and obvious involvement of other systems in addition to common respiratory symptoms, with a high mortality rate. This study summarizes the nursing experience of a patient with severe pneumonia complicated with multiple disorders under a multidisciplinary team, aiming to provide a more comprehensive theoretical and practical reference for the nursing of patients with severe pneumonia complicated with multiple disorders.

**Patient concerns:**

The patient, a 43-year-old male, was admitted to the hospital due to fever for 2 days and dyspnea for 6 hours.

**Diagnoses:**

The patient was admitted to intensive care unit (ICU) as severe pneumonia.

**Interventions:**

The key points of nursing included extracorporeal membrane oxygenation (ECMO) management, sequential anticoagulation, reasonable sedation and analgesia, prevention and control of multi-drug resistant bacteria, nutritional support and gastrointestinal reaction management, staged rehabilitation training and humanistic care.

**Outcomes:**

After 70 days of active treatment and careful nursing, the patient was successfully cured and discharged from the department for rehabilitation treatment. The patient returned to society in June 2024 and joined the ranks of directors again. A micro-film he directed and acted in will be released nationwide this year.

**Lessons:**

First, early recognition and timely intervention of severe pneumonia are essential. Second, in the face of complex disease, multidisciplinary team cooperation is conducive to improve the accuracy of diagnosis and the effectiveness of treatment. Third, it is very necessary to do a good job in family management. Last, the study of new business and technology should be strengthened in the future to improve the professional ability of medical staff and the treatment level of critically ill patients.

## 1. Introduction

Severe pneumonia is an acute severe respiratory disease caused by pulmonary infection, often accompanied by severe hypoxemia and systemic inflammatory response syndrome.^[1]^ Due to the spread of infection and inflammation, patients are prone to progress to multiple organ dysfunction syndrome and have a high mortality rate.^[2]^ Therefore, early recognition and timely intervention of severe pneumonia are essential. In January 2024, a patient with severe pneumonia was admitted to our department, and he was subsequently complicated with multiple disorders. After 70 days of active treatment and careful nursing by the multidisciplinary team, the patient was successfully treated and discharged for rehabilitation treatment. The key points of nursing are reported as follows.

## 2. Case report

### 2.1. Ethical issue

The written informed consent was obtained from the patient. And this case report has been approved by the Ethics Committee of Xiangyang First People’s Hospital Affiliated to Hubei University of Medicine with approval No.XYYYE20240108.

### 2.2. General information

The patient, a 43-year-old male, was admitted to the hospital on January 9, 2024, due to fever for 2 days and dyspnea for 6 hours. The patient had a history of hypertension for 7 years and had taken irbesartan and amlodipine to control blood pressure for a long time. He denied the history of other major diseases and had no history of allergies. During the emergency period, the patient’s consciousness gradually became blurred, and he was given endotracheal intubation and ventilator assisted breathing. The patient’s chest CT showed bilateral lung infection, and he was admitted to our department as severe pneumonia. On admission, the patient’s heart rate was 110 beats/min, sinus rhythm was normal, blood pressure was 123/79 mmHg, respiration was 20 beats/min, pulse oximetry oxygen saturation (SpO_2_) was 79%, bilateral pupil diameter was 3.0 mm, and light reflex was weakened. The rhythm was consistent, the heart sounds were relatively loud, no murmur was heard, the breath sounds of both lungs were low, the abdomen was soft, no bowel sounds were heard, and there was no edema in both lower limbs. Blood gas analysis showed that PH was 7.22, PCO2 55 mm Hg, PO2 58 mm Hg, BE 5.2 mmol/L, blood glucose 10 mmol/L, lactate 3.1 mmol/L, hematocrit 55%, potassium 3.6 mmol/L, ionized calcium 1.04 mmol/L, and sodium 134 mmol/L.

### 2.3. Treatment and outcome

Emergency venovenous extracorporeal membrane oxygenation implantation was performed on the day of admission due to severe pneumonia and respiratory failure. The rotational speed was maintained at 2500 to 2857 RPM, the blood flow at 3.5 to 4.2 L/min and gas flow at 3.4 to 3.8 L/min. Continuous venovenous hemodiafiltration was applied due to anuria, increased serum creatinine, sepsis, severe capillary leakage, and systemic edema during ECMO support. On January 10, 2 additional hemoperfusion procedures were performed with model numbers HA330 and HA380. On January 16, the patient was weaned from ECMO successfully. On February 7, the patient had no indication for continuous renal replacement therapy (CRRT). From January 9 to 17, critical values for troponin I, activated partial thromboplastin time (APTT), and white blood cell count were reported repeatedly. From January 11 to February 20, methicillin-resistant staphylococcus aureus, acinetobacter joni, burkholderia neocepacia, aspergillus, and carbapenem-resistant *Acinetobacter baumannii* were detected in sputum cultures. Blood cultures revealed staphylococcus aureus, burkholderia neocepacia, methicillin-resistant coagulase-negative staphylococcus, and *Sphingomonas paucimobilis*. On January 21, the patient developed a rash all over the body. Following the advice of dermatology consultation, the patient was given nasal feeding with cetirizine and external application of calamine. From January 22 to discharge, the patient had frequent diarrhea and received nasal feeding of montmorillonite powder and tetraplex viable bacteria tablets according to the doctor’s advice. After consultation with traditional Chinese medicine physicians, Chinese medicine was added for invigorating spleen, eliminating dampness, clearing heat and antidiarrhea. On January 24, the patient could open his eyes and nod slightly when called her, and the rash all over the body improved. On January 26, the patient could open her eyes spontaneously and had grade II muscle strength in both limbs. On February 2, the patient was able to communicate simply, the muscle strength of both upper limbs was grade IV, and the muscle strength of both lower limbs was grade II, and the patient was treated with rehabilitation. On February 29, the patient received oral enteral nutrition supplemented with parenteral nutrition. On March 4, the patient developed anxiety, and drug intervention and psychological intervention were given. On March 7, the patient was gradually weaned from the ventilator to exercise his respiratory function. On March 16, the patient took oral food and the nasointestinal tube was removed. CT showed (1) Multiple bronchiectasis and patchy high-density shadows in both lungs, considering infectious lesions. The left lower lobe of the lung was consolidated, which was better than before, but the consolidation of the right lower lobe was not significantly changed; (2) Bilateral pleural thickening and adhesion without bilateral pleural effusion; (3) Gallbladder enlargement with slightly increased gallbladder density; (4) There was no significant change in the patchy low density shadow in the liver; (5) Abdominal pneumatosis.

On March 17, laboratory tests showed that the patient’s white blood cell was 8.56 × 10^9^/L, red blood cell 3.73 × 10^12^/L, hemoglobin 121 g/L, platelet 381 × 10^9^/L, absolute neutrophil 4.67 × 10^9^/L, absolute lymphocyte 2.93 × 10^9^/L, absolute monocyte 0.78 × 10^9^/L, absolute eosinophil 0.12 × 10^9^/L, absolute basophil 0.06 × 10^9^/L, hematocrit 0.378, mean corpuscular volume 101.3 fL, mean corpuscular hemoglobin 32.4 pg, mean corpuscular hemoglobin 320 g/L, red blood cell volume distribution width 16.5%, red blood cell distribution width 61.1%, mean platelet volume 9.7 fL, platelet specific volume 0.37%, platelet distribution width 10.1%, and large platelet ratio 21.6%. The percentage of neutrophil, lymphocyte, monocytes, eosinophils, and basophils were 54.6%, 34.2%, 9.1 %, 1.4 %, and 0.7 %, respectively.

On March 18, the patient’s heart rate ranged from 100 beats/min to 110 beats/min, respiration was 23 beats/min, blood pressure was 145/72 mmHg, SpO_2_ was 96%. The patient’s body temperature was normal, the breath sounds of both lungs were thick, the rhythm was consistent, the abdomen was soft, the bowel sounds were audible, and there was no obvious abdominal pain and distension. Laboratory tests showed that potassium was 4.26 mmol/L, sodium 139.3 mmol/L, chloride 109.0 mmol/L, total calcium 2.29 mmol/L, inorganic phosphorus 1.26 mmol/L and magnesium 0.75 mmol/L. The chyle index, hemolysis index and jaundice index were all negative. The multidisciplinary team adjusted the treatment plan according to the patient’s daily situation in real time. After 70 days of active treatment and careful nursing, the patient was treated in the department of rehabilitation on March 18. The patient returned to society in June 2024 and joined the ranks of directors again. A micro-film he directed and acted in will be released nationwide this year.

## 3. Nursing care

### 3.1. Standardize ECMO management

ECMO equipment management is a complex process that requires professional medical staff, strict operating procedures, and continuous quality control to ensure patient safety and treatment outcomes.^[3]^ Therefore, we carried out a series of effective nursing interventions: ①Vascular access management: To ensure that the pipeline is unobstructed, avoid distortion or displacement, and prevent thrombosis; Keep the catheter place clean and dry, change the dressing regularly and fix it properly; Heparin dose was adjusted by regularly monitoring coagulation function. ②Respiratory and circulatory support: Respiratory specialist nurses and CRRT specialist nurses regularly monitored the mode and parameters of mechanical ventilation and CRRT, and adjusted them in real time to adapt to the changes during ECMO treatment. ③Complication prevention: Tissue oxygen saturation monitor was used to monitor the SpO_2_ level of the patient’s limbs on the catheterization side, and the skin ecchymosis and cyanosis were observed to prevent hypoxic-ischemic injury caused by hypoperfusion. The patient was bedridden for a long time, assisted by air cushion bed and foam dressing, and turned over every 2 hours for the patient to effectively prevent the occurrence of pressure injury.^[4]^ Low molecular weight heparin subcutaneous injection, nasal feeding warfarin, lower limb elevation, and pneumatic pressure therapy were used to promote lower limb blood circulation, reduce swelling, prevent deep vein thrombosis and improve lower limb venous function. ④Equipment monitoring and maintenance: The nurses in the monitoring shift carried out quality control every day, the nurses in the computer shift carried out quality reporting, and the responsible nurses carried out rectification to ensure that all the equipment worked normally and the parameters were within the normal range. During ECMO support, the right limb of the patient had good blood supply, and no adverse events occurred. During weaning, the patient’s circulation was stable and overall progress was stable.

### 3.2. Anticoagulation and bleeding

Anticoagulation is a key link in ECMO and CRRT treatment.^[5]^ Since the patient was admitted to our department, the APTT had been reported as critical value for many times, and the value was more than 150 seconds. The patient had epistaxis, bleeding around the ECMO puncture site, and DIC. During ECMO support, heparin sodium was continuously infused through a syringe pump with an initial dose of 10 mg/h, and the subsequent dose was maintained at 4 to 10 mg/h. During CRRT, 4% sodium citrate was continuously pumped through an infusion pump, the initial dose was 200 mL/h, and the subsequent dose was maintained at 200 to 220 mg/h. The target range of APTT and activated clotting time was set at 50 to 60 seconds and 180 to 200 seconds, respectively, and the monitoring frequency was 2 and 8 h, respectively. The pumping speed of heparin sodium and sodium citrate was dynamically adjusted. For the patient with epistaxis and bleeding around ECMO puncture sites, the expanded sponge was used to compress the nasal cavity to stop bleeding according to the doctor’s advice, and fresh frozen plasma was infused to supplement coagulation factors at the same time. The dressing at the catheter site was replaced and properly fixed according to the situation, and the responsible nurses focused on the observation. From January 17 until the patient discharged from our department, the APTT critical value was not reported again.

### 3.3. Reasonable sedation and analgesia

Sedation and analgesia management of critically ill patients is an important part of treatment, which is a dynamic process. It is necessary to adjust the treatment plan in time according to the changes of the patient’s condition, so as to reduce the pain and anxiety of patients and ensure the safety of treatment operations.^[6]^ In order to ensure the effectiveness of sedation and analgesia, our nursing focus was as follows: The richmond agitation–sedation scale and intensive care pain observation tool were used to evaluate the patient, and the patient’s consciousness level, agitation state and pain degree were monitored in real time. If the patient is agitated or appears to be resistant to the ventilator, inform the doctor and adjust the intravenous infusion rate of midazolam, propofol or dexmedetomidine or adjust the ventilator parameters to maintain the best ventilatory status of the patient. At the same time, remifentanil or sufentanil were used to control the pain of the patient and improve his tolerance to mechanical ventilation. Bispectral index (BIS) was used to continuously monitor the sedation depth of the patient to ensure that BIS score was maintained at about 50 to 60 points to prevent respiratory depression or even disturbance of consciousness caused by excessive sedation.^[7]^ Health education was given to the patient when he was awake and could communicate, and the effects and side effects of sedative and analgesic drugs were explained to the patient to prevent the formation of drug dependence. The patient was given protective restraint during moderate sedation and analgesia. After the patient was awake and able to communicate effectively, the protective restraint was removed after the full evaluation of the medical staff. No adverse events such as unplanned extubation occurred during the period.

### 3.4. Prevention and control of multi-drug resistant bacteria

In view of the complicated infection situation of the patient, the clinical team formulated the corresponding prevention and control measures: The patient was placed in the isolation ward to prevent cross infection. The ward was equipped with an independent air filtration system to ensure air circulation and purification. The isolation sign was hung at a prominent position beside the patient’s bed, and isolation gown, isolation mask and polyvinyl chloride gloves were provided. Health care workers are required to wear isolation gown and polyvinyl chloride gloves before touching the patient, while performing any invasive procedures, and when touching body fluids, secretions, or excreta of the patient. Isolation gowns are usually changed daily, and new gowns are replaced as soon as contamination or dampness is detected. Medical staff should use quick-drying hand disinfectant to disinfect their hands when hand hygiene indications appear. Thoroughly clean and disinfect all objects in the ward every day, and separate disinfect items and special equipment used by the patient. At the same time, the microbial monitoring of the ward environment was carried out regularly to evaluate the disinfection effect, and possible contamination points were found and treated in time. Strictly deal with medical waste, sharp instruments are placed in sharp boxes, and other medical waste is placed in double-layer yellow garbage bags, and transported to the temporary storage of medical waste in the hospital. Specimens were transported in leak-proof airtight containers. Closely monitoring the infection indicators of the patient, including body temperature, blood routine, sputum culture and blood culture results. On February 5, the patient’s sputum and blood cultures showed methicillin-resistant staphylococcus aureus decolonization.

### 3.5. Nutritional support and gastrointestinal reaction management

In order to ensure the nutritional supply of the patient, we adopted enteral nutrition, parenteral nutrition and central venous high nutrition support. Since January 21, the patient had diarrhea for many times, and the stool was yellow and thin, with a large amount. According to the doctor’s advice, the patient received nasal feeding of montmorillonite powder and tetralogy of live bacteria tablets. After consultation with traditional Chinese medicine physicians, Chinese medicine was added for invigorating spleen, eliminating dampness, clearing heat and antidiarrhea. On February 23, the weight of the patient decreased and returned to 90 kg when he left our department. In order to ensure the nutritional needs of the patient and effectively deal with the gastrointestinal reactions of the patient, the nursing focus of nutrition specialist nurses is as follows: Assist doctors to formulate nutritional treatment plan, and adjust the nutritional formula and dose according to the patient’s tolerance. Keep the nasogastric tube and nasointestinal tube unobstructed to prevent blockage. The nasal feeding volume and tolerance of the patient were monitored and recorded, and the 4-hour gastric residual volume was determined by ultrasound. Through the modified Watian drinking test, it was judged that the patient could still eat through the mouth even if the tracheotomy and nasogastric tube were placed. Ice stimulation method was used to place ice at the base of the patient’s tongue, and lemon water was sprayed into the mouth to stimulate the patient’s taste buds and improve the patient’s appetite. The frequency of diarrhea, the character and quantity of feces were closely monitored, and the occurrence of IAD was prevented by using OB built-in sanitary pads and indwelling balloon anal tube. Explain the causes of diarrhea, treatment methods and the importance of nutritional support to the patient and his family members, and guide family members how to match diet for the patient. The nurses in the treatment shift took oral care for the patient twice a day, and the nurses in charge turned the patient over every 2 hours. The patient was cleaned in time to keep the hip skin dry, the foam dressing was replaced as appropriate, and the air cushion bed was used for decompression to prevent skin damage. After the joint efforts of doctors, nurses, the patient and his family members, the patient’s diarrhea was significantly improved on February 14, and he began to take homogenate diet orally and gradually added enteral nutrition. On March 16, the nasointestinal tube was removed.

### 3.6. Implementation of staged rehabilitation training

#### 3.6.1. Early motor rehabilitation

After gradually reducing the use of sedative and analgesic drugs, on February 3, the patient was conscious, able to nod and gesture, and had voluntary movement of both limbs. The muscle strength of both upper limbs was grade IV, and the muscle strength of both lower limbs was grade II. The patient was assisted with rehabilitation treatment such as muscle strength training, joint mobilization training, and spinal correction at the bedside, and encouraged the patient to actively move to prevent muscle atrophy and joint stiffness caused by long-term bed rest.

#### 3.6.2. Pulmonary rehabilitation

After weaning from ECMO on January 16, the patient developed shortness of breath and respiratory resistance. According to the doctor’s advice, the ventilator mode was adjusted to bi-level positive pressure ventilation and deepened sedation and analgesia, and the head of the bed was routinely elevated 30 to 45 degrees. The patient underwent a tracheotomy on January 25. Since February 7, the patient’s consciousness was clear. The respiratory specialist nurse guided the patient to perform deep breathing and cough exercises, undergo external diaphragm pacing therapy every day, carry out high flow humidification oxygen therapy for about 9 hours in the daytime, and ventilator assisted breathing at night to exercise respiratory function.

### 3.7. Humanistic care

The patient had been in the intensive care unit (ICU) for a long time, and was under sedation and analgesia for a long time due to the complex condition and treatment needs. When the patient gradually recovered from the deep sedation, the medical staff first evaluated the patient’s cognitive function, explained the current situation to the patient, closely observed the patient’s expression changes and physical activity, and helped the patient gradually adapt to the awake state and reduce his discomfort through patient communication, appropriate environmental adjustment and necessary analgesic measures. The specific measures were as follows: After the patient was admitted to ICU, the patient’s sleep pattern, psychological state, living habits and personality traits were mastered by family members, and the shift was completed to ensure that the nurses in each shift could fully grasp the patient’s information so as to carry out personalized nursing. The ward was equipped with a clock, and the orientation of time, place and person was strengthened during the shift to help the patient establish the concept of day and night time. Using blackout curtains to reduce the direct exposure of natural light, adjusting indoor lighting at night, ensuring that the light source of medical equipment is turned off or adjusted to the lowest brightness when not in use, reducing unnecessary light stimulation, and providing the patient with eye masks and earplugs to promote patient’s sleep. The patient and his family members were arranged to meet on WeChat video twice a week, and the duration of each meeting was controlled to 10 to 15 minutes. By sharing each other’s recent situation, care and support were passed, so as to encourage each other and enhance the confidence of the patient to overcome the disease and the accompanying power of his family members. Music therapy was used as an auxiliary means of drug therapy. The patient was equipped with wireless headphones, and according to his music preferences, music was played every morning when he woke up and at night before he fell asleep, for 30 minutes each time, to relieve his anxiety. When the patient wakes up, he knows his condition and believes that there is no hope for recovery, falling into a negative lying state. The psychological specialist nurses apply narrative therapy to help the patient find the courage and hope of life again. The patient rekindle his enthusiasm for micro-film production, and returns to society in June 2024 to join the line of directors again. The micro-film he directs and takes part in will be released nationwide this year.

## 4. Limitations

The limitations of this study mainly include the single sample size, the potential bias of retrospective study, the complexity of multidisciplinary team collaboration, and the subjectivity of nursing intervention. These factors may affect the broad applicability and objectivity of the findings. In order to cope with these challenges, future research can expand sample size, optimize data management, strengthen multidisciplinary collaboration, standardize nursing intervention, carry out long-term follow-up, improve resource support, and strictly abide by ethical norms to further improve the scientific nature and practicability of research, and provide a more comprehensive theoretical and practical basis for the nursing of patients with severe pneumonia complicated with multiple disorders.

## 5. Summary

The patient’s condition is complex and diverse, and the treatment measures are diverse and frequently adjusted, which requires the medical team to have accurate judgment and flexible coping strategies to ensure that the patient receives timely and effective treatment and care. Reflection of this case: Early recognition and timely intervention of severe pneumonia are essential. In the face of complex disease, multidisciplinary team cooperation is conducive to improve the accuracy of diagnosis and the effectiveness of treatment. The patient was hospitalized in ICU for a long time due to his critical condition, medical staff should pay attention to effective communication with family members and do a good job in family management. The study of new business and new technology should be strengthened in the future to improve the professional ability of ICU medical staff and the treatment level of critically ill patients.

## Author contributions

**Conceptualization:** Zhenzhen Yuan, Yinhao Yang, Qun Liu, Bolun Zhang, Xingxing Wang, Shiqun Huang, Wenli Ma.

**Data curation:** Zhenzhen Yuan, Yinhao Yang, Qun Liu, Bolun Zhang, Xingxing Wang, Shiqun Huang, Wenli Ma.

**Investigation:** Yinhao Yang, Qun Liu.

**Project administration:** Zhenzhen Yuan.

**Resources:** Zhenzhen Yuan.

**Supervision:** Zhenzhen Yuan.

**Writing – original draft:** Zhenzhen Yuan, Yinhao Yang, Qun Liu.

**Writing – review & editing:** Zhenzhen Yuan, Yinhao Yang, Qun Liu, Bolun Zhang, Xingxing Wang, Shiqun Huang, Wenli Ma.
